# A method for measuring the molecular ratio of inhalation to exhalation and effect of inspired oxygen levels on oxygen consumption

**DOI:** 10.1038/s41598-021-91246-8

**Published:** 2021-06-17

**Authors:** Koichiro Shinozaki, Yu Okuma, Kota Saeki, Santiago J. Miyara, Tomoaki Aoki, Ernesto P. Molmenti, Tai Yin, Junhwan Kim, Joshua W. Lampe, Lance B. Becker

**Affiliations:** 1 The Feinstein Institutes for Medical Research, Northwell Health, Manhasset, NY USA; 2 Nihon Kohden Innovation Center, Cambridge, MA USA; 3 Elmezzi Graduate School of Molecular Medicine, Manhasset, NY USA; 4 ZOLL Medical, Chelmsford, MA USA; 5 Department of Surgery, Medicine, and Pediatrics, Zucker School of Medicine at Hofstra/Northwell, Hempstead, NY USA; 6 Department of Emergency Medicine, Zucker School of Medicine at Hofstra/Northwell, Hempstead, NY USA; 7 Department of Emergency Medicine, North Shore University Hospital/Long Island Jewish Medical Center, Northwell Health, 300 Community Dr., Manhasset, NY 11030 USA

**Keywords:** Cardiovascular models, Respiratory system models, Cardiovascular biology, Applied physics, Biomedical engineering

## Abstract

Using a new method for measuring the molecular ratio (R) of inhalation to exhalation, we investigated the effect of high fraction of inspired oxygen (FIO2) on oxygen consumption (VO2), carbon dioxide generation (VCO2), and respiratory quotient (RQ) in mechanically ventilated rats. Twelve rats were equally assigned into two groups by anesthetics: intravenous midazolam/fentanyl vs. inhaled isoflurane. R, VO2, VCO2, and RQ were measured at FIO2 0.3 or 1.0. R error was ± 0.003. R was 1.0099 ± 0.0023 with isoflurane and 1.0074 ± 0.0018 with midazolam/fentanyl. R was 1.0081 ± 0.0017 at an FIO2 of 0.3 and 1.0092 ± 0.0029 at an FIO2 of 1.0. There were no differences in VCO2 among the groups. VO2 increased at FIO2 1.0, which was more notable when midazolam/fentanyl was used (isoflurane-FIO2 0.3: 15.4 ± 1.1; isoflurane-FIO2 1.0: 17.2 ± 1.8; midazolam/fentanyl-FIO2 0.3: 15.4 ± 1.1; midazolam/fentanyl-FIO2 1.0: 21.0 ± 2.2 mL/kg/min at STP). The RQ was lower at FIO2 1.0 than FIO2 0.3 (isoflurane-FIO2 0.3: 0.80 ± 0.07; isoflurane-FIO2 1.0: 0.71 ± 0.05; midazolam/fentanyl-FIO2 0.3: 0.79 ± 0.03; midazolam/fentanyl-FIO2 1.0: 0.59 ± 0.04). R was not affected by either anesthetics or FIO2. Inspired 100% O2 increased VO2 and decreased RQ, which might be more remarkable when midazolam/fentanyl was used.

## Introduction

Oxygen consumption (VO2), carbon dioxide generation (VCO2), and respiratory quotient (RQ), which is the ratio of VCO2 to VO2, are essential measures of metabolism^1,2^. For more than 100 years, investigators have been exploring methods of measuring VO2 and VCO2^3^ and much research in recent years has focused on their clinical applications^4,5^. Indirect calorimetry is a non-invasive method, in which VO2 and VCO2 are measured from inhalation and exhalation of a subject^6–8^. Since it is non-invasive, indirect calorimetry has been widely used in clinical research^9,10^ and translational science^11–13^. However, although the basic principles of indirect calorimetry are well established, there are critical pitfalls in its methodology that must be appreciated^8,14,15^.

One of the central problems is a volume/flow measurement of inhalation and/or exhalation. Errors are propagated when the volume measurement for both inhalation and exhalation are inaccurate^8,13^. Therefore, Haldane transformation, by which a volume of inhalation is estimated from that of exhalation, has been investigated and accepted over the last decades^16,17^. However, its utility is limited, especially when the fraction of inspired oxygen (FIO2) increases to more than 0.6, because the denominator of Haldane transformation goes to zero when 100% oxygen is inspired. And so, few studies to date have focused on the effect of FIO2 on metabolism^18^.

There have been debates about the effect of high FIO2 on oxygen metabolism. Lodato (1989)^19^ monitored oxygen consumption during normobaric hyperoxia. In this study, decreased VO2 was reported at hyperoxia in dogs with a Fick method, by which the amount of oxygen in the blood circulation and the cardiac output were used to calculate VO2. However, Chapler (1984)^20^ found no effect of hyperoxia on VO2 under control conditions. None of these studies using the Fick method reported VCO2 or RQ and so an important piece of the metabolic information was missed. Therefore, Lauscher (2012)^18^ investigated the effect of high FIO2 on metabolism and used indirect calorimetry with their modified Haldane transformation and showed increased VO2 and decreased RQ at an FIO2 of 1.0. However, the modified transformation has not been widely accepted and its accuracy remains unclear. As a result, it is not clear yet whether and how 100% inspired oxygen affects oxygen metabolism and/or VCO2 and RQ.

Commercially available gas flow sensors have errors ranging from 3 to 5%, which can increase errors of VO2 by 60–100% when FIO2 1.0 is used. The present paper demonstrates a new method for measuring the molecular ratio of inhalation to exhalation, which is approximately 10 times more accurate than commercially available sensors. Unlike Haldane transformation, our method is an independent measurement and does not use estimation from gas concentrations. Our measurement of the molecular ratio allows for further investigations. The present investigation was undertaken to determine to what extent, if any, an FIO2 of 1.0 influences the values of VO2, VCO2, and/or RQ in mechanically ventilated rodents. We also investigated the effect of an FIO2 of 1.0 on the molecular ratio of inhalation to exhalation. In addition, we explored factors that could influence the molecular ratio, a critical variable that affects values of VO2 and RQ.

## Materials and methods

The Institutional Animal Care and Use Committees (IACUC) of the Feinstein Institute for Medical Research approved these study protocols. All experiments were performed in accordance with relevant guidelines and regulations and this study is reported in accordance with ARRIVE guidelines. The data supporting this study are available from the corresponding author upon reasonable request. One investigator performed all surgical procedures; therefore we did not apply blinding procedures.

### Animal preparation

**Figure 1:**
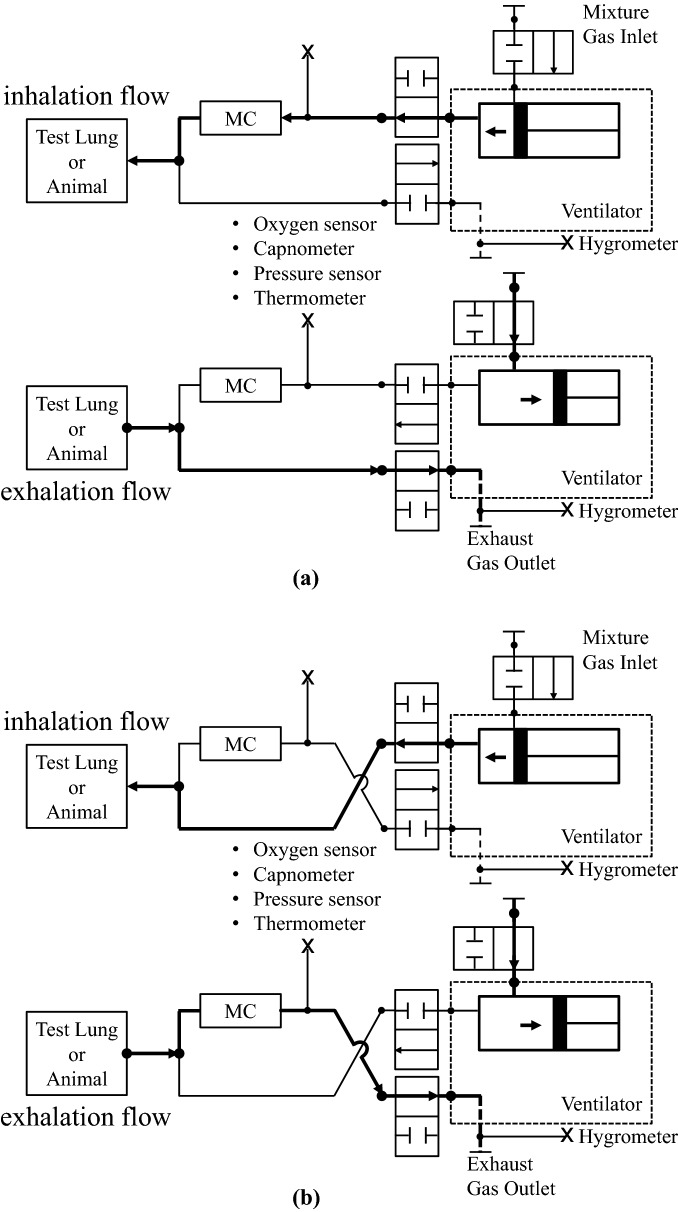
Diagram of the mechanical ventilation circuit. (**a**) Before and after oxygen consumption measurements, FIO2 is measured for at least ten minutes. (**b**) During measurements of oxygen consumption, FEO2 is measured after the ventilator circuit has been switched. O2 extraction is calculated by a subtraction between FIO2 and FEO2. MC indicates mixing chamber.

We added some modifications to the procedures that we previously described^21^. Adult male Sprague–Dawley rats (400–500 g, Charles River Laboratories, Wilmington, MA) underwent general anesthesia with 4% isoflurane (Isosthesia, Butler-Schein AHS, Dublin, OH, USA) and were intubated by a 14-gauge plastic catheter (Surflo, Terumo Medical Corporation, Somerset, NJ). Rats underwent volume control mechanical ventilation (Ventilator Model 683, Harvard Apparatus, Holliston, MA, USA). The trachea was ligated after the endotracheal intubation in order to avoid gas leakage through the vocal cords. We fixed the minute ventilation volume (MVV) at 180 mL/min and set the respiratory rate at 45 breaths per minute. We did not change MVV or respiratory rate during experiments. Positive end-expiratory pressure (PEEP) was set at 2 cm. Carbon dioxide (CO2) was continuously measured inline in the exhalation branch of the ventilator circuit by using a CO2 gas monitor (OLG-2800, Nihon Kohden Corp., Tokyo, Japan) with a CO2 sensor (TG-970P, Nihon Kohden Corp., Tokyo, Japan) and an airway adapter (YG-211T, Nihon Kohden Corp., Tokyo, Japan). The CO2 sensor was a main-stream capnometer, which did not require any sampling volume of the gas. We focused on preventing leakage or contamination of the gas. Therefore, we chose Viton—a synthetic, less absorbable and permeable rubber—as the tubing material for our ventilator circuit and it was checked every experiment for leakage (Fig. 1). Our circuit included a 10.5 mL chamber in one side of the circuit. This chamber enabled each stroke of exhalation to be mixed in the circuit in order that the concentration of a flowing gas was averaged in the gas circuit. The fraction of expired CO2 (FECO2) was monitored real-time and we maintained values within a range of 30–45 mmHg during the surgical preparation. The core temperature (T-type thermocouple probes, ADInstruments, Colorado Springs, CO, USA) was monitored in the esophagus and it was maintained at 37 ± 0.5 °C. We placed a sterile polyethylene-50 catheter in the left femoral artery (FA) for continuous arterial pressure monitoring (MLT844, ADInstruments; Bridge Amplifier ML221, ADInstruments, Colorado Springs, CO, USA) and another catheter cannulated in the left femoral vein, which was advanced to the inferior vena cava for intra-venous drug administration.

### Measurement preparation

We used a photoluminescence-quenching sensor (FOXY AL300 Oxygen Sensor Probe, Ocean Optics, Dunedin, FL, USA) and a fluorometer (NEOFOX-GT, Ocean Optics, Dunedin, FL, USA) to measure the concentration of oxygen gas. We first checked the accuracy of the system by using 4 different gas mixtures (4% CO2 was mixed with 10%, 45%, 80%, and 96% O2; MESA International Technologies, Inc. Santa Ana, CA, USA).

For the accuracy of CO2 measurement, we evaluated the effect of 4 different O2 concentrations on the CO2 sensor and developed an equation to correct the CO2 errors. The CO2 concentration measurement decreased linearly as the O2 concentration increased. Therefore, we derived the following empirical equation:

1 $${\text{CO2}} = {\text{measured}}\;{\text{CO2}}/\left( {1 - 0.00072 \times {\text{measured}}\;{\text{O2}}} \right).$$

We performed 2-point calibration for the O2 sensor by medical air (20.9% O2 balanced with Nitrogen) and medical O2 (100% O2, General Welding Supply Corp., Westbury, NY, USA) before each experiment. The CO2 sensor was calibrated with medical air (0% CO2) and industrial CO2 (10.4% CO2 balanced with Nitrogen, General Welding Supply Corp., Westbury, NY, USA). We measured gas humidity inline with a hygrometer (TFH 620, ebro, Ingolstadt, Germany). Both calibrations occurred at 0% humidity. A temperature probe (T-type thermocouple probes, ADInstruments Inc., CO, USA) and a pressure probe (MLT844, ADInstruments; Bridge Amplifier ML221, ADInstruments, Colorado Springs, CO, USA) were placed inline. In addition, we monitored the ambient temperature and the pressure to check the condition around the ventilator system (Traceable Workstation Digital Barometer, Fisher Scientific, NH, USA).

We used a commercially available capnography system and following is the specification: measurement range, 0–20% (0–150 mmHg); accuracy, ± 2 mmHg; respiratory rate range, 0–150 breath/min. This included a main-stream component, which response time is much faster than that of a side-stream component that requires a sampling tube, creating a dead space and slowing down the response time. We used a ventilation setting of 45 breath/min, which allowed for approximately 670 ms for expiration. The mechanical ventilation circuit included a 10.5 mL chamber that enabled the mixture of expiration gas, and decreased and flattened the fluctuation of gas concentrations in expiration.

### Methods for measuring the molecular ratio of inhalation to exhalation

**Figure 2:**
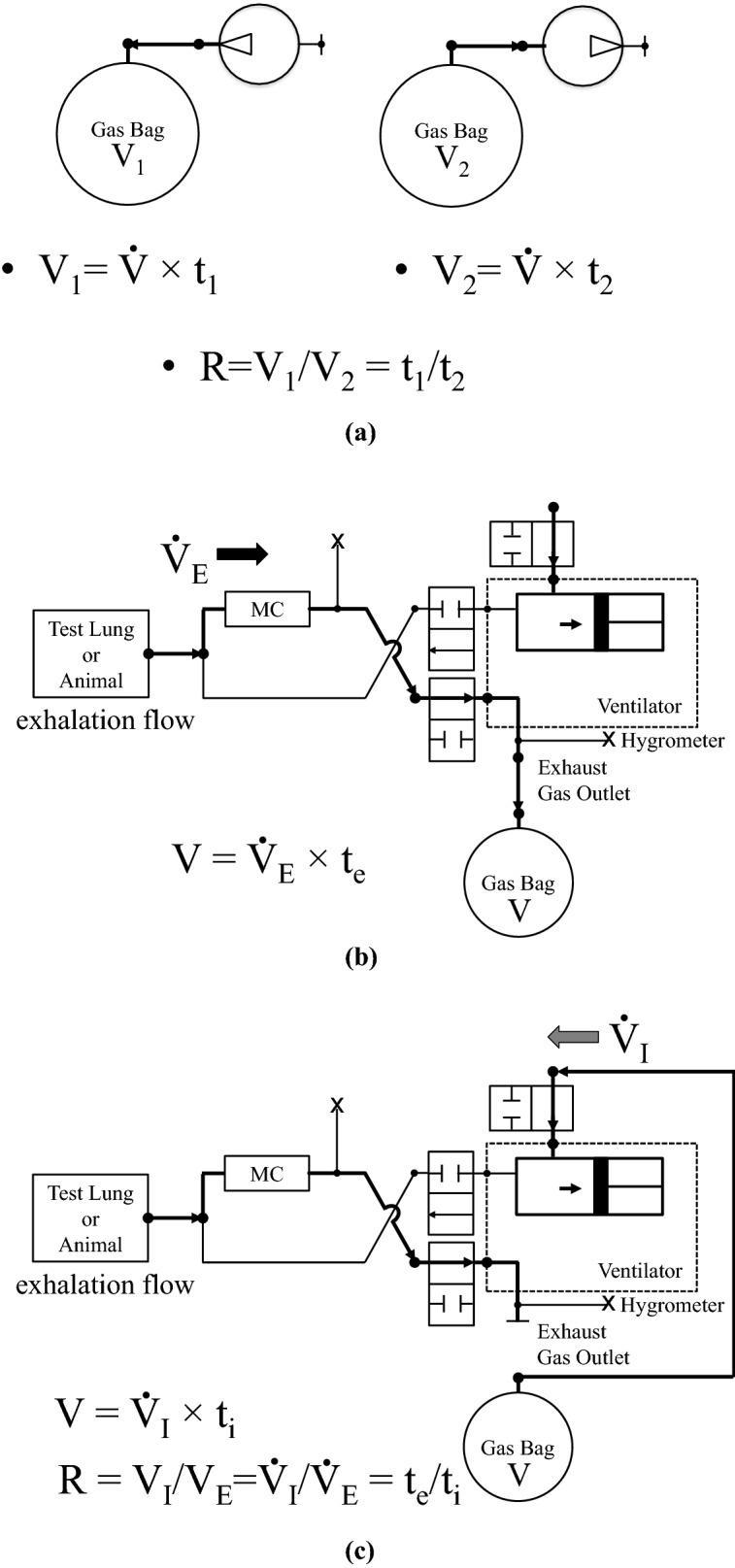
Diagram of the concept of measuring the molecular ratio of inhalation to exhalation. (**a**) V1 and V2 are the volumes in the bags containing molecules that are compared. ${\dot{\text{V}}}$ is a flow rate that is precise and consistent over the measurement driven by a gas pump, and t1 and t2 are the time to collect a gas into or to pump out a gas from the bags. In order to accurately measure the molecular ratio between V1 and V2, the temperature and the pressure of the gases are equilibrated based on the ideal gas law. The gas pump and the connected circuit need to be the same when measuring the ratio of two different volumes. (**b**) While an animal breathed a gas out, the gas is collected into a non-permeable soft gasbag. A mechanical ventilator resembling a piston system is the gas pump that precisely pumps in and out the gases that are compared. The ventilation circuit separates the animal’s inhalation and exhalation with valves in synchrony of locomotion of the piston cylinder. The exhalation flow rate is expressed as ${\dot{\text{V}}}_{{\text{E}}}$ and the time for the gas collection (t_e_) is counted. (**c**) A stopcock is closed at the end of the gas collection and the gasbag is repositioned to an inlet of the piston cylinder. The cylinder then starts pumping the gas into the circuit. The inhalation flow rate is expressed as ${\dot{\text{V}}}_{{\text{I}}}$ and time (t_i_) until the cylinder pump empties the gasbag is counted. Since the volume in the gasbag is identical expressed V in the diagram with no gas leak or penetration, the equation for R measurement is derived and shown in the figure.

We directly measured the molecular ratio of inhalation to exhalation named “R” in this paper, rather than volume measurements for inhalation and exhalation. The required components are a gasbag, a gas pump precisely working for the duration of the measurement, and a gas circuit containing valves that separate inhalation and exhalation. Figure 2 shows the concept of our measurement. V1 and V2 in Fig. 2a are the volumes in the bags containing molecules that we compare, V̇ is a flow rate that is consistent over the measurement driven by the gas pump, and t1 and t2 are the time to collect a gas into or to pump out a gas from the bags. In order to accurately measure the molecular ratio between V1 and V2, the temperature and the pressure of the gases need to be equilibrated during the measurement based on the ideal gas law. We monitored these values during the experiment and verified that they were consistent and equilibrated. If they were not, it was considered a technical error and the data was dismissed. For the precise work of the gas pump, the resistance of the gas circuit needs to be the same in both directions of the gas flow. V1 and V2 are considered inhalation and exhalation in vivo experiments.

In our experimental setting, while an animal breathed a gas out, which was exhalation (Fig. 2b), the gas was collected into a non-permeable soft gasbag (Dual-valve Kynar PVDF bag, Cole-Parmer, IL, USA). We used a mechanical ventilator resembling a piston system (Model 683, Harvard Apparatus, MA, USA). The ventilator had a short cylinder and, while it was pumping in and out against a gas, the ventilation circuit separated the animal’s inhalation and exhalation with valves in synchrony of locomotion of the piston cylinder. The exhalation flow rate was expressed as ${\dot{\text{V}}}_{{\text{E}}}$ and the time for the gas collection (t_e_) was counted. The volume of the gas collected in the gasbag or ${\dot{\text{V}}}_{{\text{E}}}$ was unknown. A stopcock was closed at the end of the gas collection and the gasbag was repositioned to an inlet of the piston cylinder. The cylinder then started pumping the gas into the circuit. The inhalation flow rate was expressed as ${\dot{\text{V}}}_{{\text{I}}}$ (Fig. 2c) and time (t_i_) until the cylinder pump emptied the gasbag was counted. ${\dot{\text{V}}}_{{\text{I}}}$ was unknown. The gasbag was prepared and emptied before starting the gas collection. A pressure sensor was attached and the gasbag was set at a negative pressure before the measurement (pressure was normally set at − 2 to − 3 mmHg). When the pressure inside the bag reached the original negative pressure, it was considered that the pump emptied the gasbag. Since the volume in the gasbag was identical with no gas leak or penetration, the following equation was derived:

2 $${\dot{\text{V}}}_{{\text{I}}} \times {\text{t}}_{{\text{i}}} = {\dot{\text{V}}}_{{\text{E}}} \times {\text{t}}_{{\text{e}}}$$

3 $${\dot{\text{V}}}_{{\text{I}}} /{\dot{\text{V}}}_{{\text{E}}} = {\text{t}}_{{\text{e}}} /{\text{t}}_{{\text{i}}}$$

The flow ratio of inhalation to exhalation was equivalent to t_e_ over t_i_. We monitored the temperature and pressure of the gas and confirmed that those inside the gasbag were equilibrated to the ambient while counting t_e_ and t_i_. Therefore, because the temperature and the pressure were constant during the measurements, the calculated number of t_e_ over t_i_ was the molecular ratio (R) of inhalation to exhalation of the animal.

As we previously reported^21^, we used an inverted, water-sealed 500 mL cylinder filled with water for the measurement of the volume of expired gas (VE). Expired gas displaced the water in the cylinder during volume measurements. We collected expired gas for 2 min and the MVV was calculated from this number. We performed the VE measurements twice in each experiment before and after the metabolic measurement and verified that these values did not change over the experiment.

### Accuracy of the molecular ratio measurements

We assembled the system that was used for the animal experiments and evaluated the accuracy of our R measurement. The mechanical ventilator was connected to a small rubber balloon (test lung) instead of an animal. The gasbag was emptied before starting the gas collection and we added a drop of deionized/filtered water inside the bag. A dry gas was collected into the bag for 10 min. The water drop was vaporized inside the bag and added a gas volume that was measured as humidity. We created a variety of humidity level adjusting for the amount of water from zero to 30 μL. We measured the temperature and the relative humidity of the gas collected in the bag. The hygrometer was attached in a closed loop circuit, which had no gas leak, and a sample gas for the humidity measurement was returned to the gasbag after the humidity measurement. We calculated the molecular ratio of a dry gas to a humid gas based on the values of temperature and relative humidity obtained from this experiment.

### Calculations and corrections

FIO2, FEO2 (fraction of expired oxygen), FICO2 (fraction of inspired carbon dioxide), FECO2, in-circuit gas pressure and temperature, relative humidity and temperature in exhalation, and ambient pressure and temperature around the ventilator circuit were measured and monitored during animal experiments (Fig. 1). Data were collected every 1 s and the average of 60 s was extracted. FEO2 and FECO2 were measured after the ventilator circuit was switched from the measurements of FIO2 and FICO2. After 60 min of the FEO2 and FECO2 measurements, the circuit was switched back and post-measurement FIO2 was recorded. The data collection of FIO2 was performed at a dry gas, so 15 min were needed to dry the circuit after switching from FEO2 to FIO2 for post-FIO2 measurement. Based on our preliminary experiments, approximately 10 min were needed to dry our circuit, which was humidified by the animal’s exhalation. The time series of dry FIO2 was calculated from the values of pre- and post-FIO2. The calibrations for the O2 and the CO2 sensors were performed under ambient pressure and so the values of the gas concentrations required standardization by the in-circuit pressure, which was 10–15 mmHg higher than the ambient pressure because of PEEP, circuit resistance, and lung compliance of the animals. We first corrected the gas concentrations with the in-circuit pressure and then the value of dry FEO2 was calculated from this number, the humidity, and the temperature of exhalation. Using the calculated value of dry FEO2 and Eq. (1), FECO2 was corrected. The correction diagram is found in supplemental data (https://figshare.com/s/cc386bff8e1e626b076e). R was measured in mechanically ventilated, anesthetized rats. We were able to calculate RQ using R in conjunction with the conventional measurement of FIO2, FEO2, and FECO2. VO2 and VCO2 were calculated by the following equations:

4 $${\text{R}} = {\text{VI}}/{\text{VE}}$$

5 $${\text{VO}}2 = {\text{VI}} \times {\text{FIO}}2 - {\text{VE}} \times {\text{FEO}}2$$

6 $${\text{VCO}}2 = {\text{VE}} \times {\text{FECO}}2 - {\text{VI}} \times {\text{FICO}}2.$$

FICO2 was zero since the inspired gas did not contain CO2. The RQ, VO2, and VCO2 were then transformed to the following equation:

7 $${\text{VO}}2 = \left( {{\text{R}} \times {\text{FIO}}2 - {\text{FEO}}2} \right) \times {\text{VE}}$$

8 $${\text{VCO}}2 = {\text{FECO}}2 \times {\text{VE}}$$

9 $${\text{RQ}} = {\text{VCO}}2/{\text{VO}}2.$$

### Experimental protocol: anesthetics and FIO2

After preparation, FIO2 was recorded with an O2 sensor at a gas circuit connected to the inhalation port of the mechanical ventilator (Fig. 1a). And then the in/out ports of the gas circuit were switched and FEO2 and FECO2 measurements were started (Fig. 1b). An initial 30 min were given for the stabilization of the animal followed by 10 min of the FEO2 and FECO2 recording. During the 30 min of the stabilization, the gas circuit and its inner condition was equilibrated with the animal’s exhalation and the relative humidity reached its maximum, which was approximately 90–93% at room temperature at the exhaustion port. After the 10-min recording, another 20 min were given for R measurements. Overall, we took 60 min for each set of the gas measurements. The gas circuit was immersed in a water bucket so that the temperature of the gas circuit was maintained. The water temperature was equilibrated to the ambient temperature.

Animals were assigned to two anesthetic protocols: intravenous anesthetics midazolam (20 mg/h/kg) and fentanyl (50 μg/h/kg); inhaled anesthetic isoflurane (2.5%). Six animals were assigned to each protocol and a total of 12 animals were enrolled in this study. For each anesthetic protocol, two different settings of FIO2 were tested: FIO2 of 0.3 and FIO2 of 1.0. Three animals were started at a FIO2 of 0.3 followed by a FIO2 of 1.0 and another 3 animals were started at a FIO2 of 1.0 followed by a FIO2 of 0.3. The interval after switching the O2 concentrations was approximately 30 min in order to equilibrate the body to the target FIO2. VO2, VCO2, and RQ were calculated thereafter accordingly.

### Experimental protocol: volume and pressure of ventilation

We anesthetized rats with isoflurane (2.5%) and changed a condition of the animal’s ventilation. MVV ranged from 150 to 300 mL/min at an FIO2 of 0.3. MVV was next set at 180 mL/min in order to maintain MVV at 8–10 mL/min/kg for the animals, and we changed the airway pressure by changing the thoracic compliance of the rats using a rubber band, which tightened the animal’s chest. The mean airway pressure inside the circuit was monitored and adjusted from 5 to 35 mmHg. The airway pressure was calculated from area under the curve of the intra-circuit pressures. R was measured at each condition of the ventilation.

### Statistical analysis

We reported data as mean and SD. Statistical analyses were performed using JMP (version 10.1 software; SAS Institute, Cary, NC, USA). Mann–Whitney U test for continuous variables was used. We considered p-value less than 0.05 as statistically significant.

## Results

### Accuracy of the molecular ratio (R) measurements

**Figure 3:**
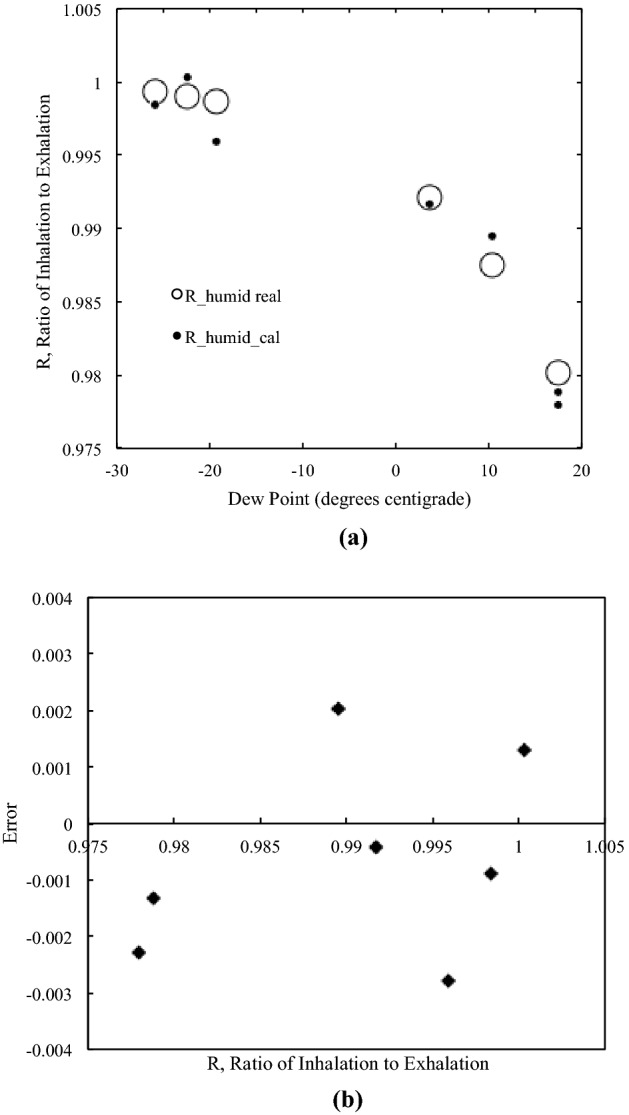
The Accuracy of R measurements. The molecular ratio of a dry gas to a humid gas was measured. The experiment was completed at room temperature (22–23 °C) and atmospheric pressure (1002–1006 hPa). (**a**) R is shown as a function of dew points calculated from the vapor pressure of water at these conditions. (**b**) Errors of the measurements were ± 0.003 (0.3%).

Figure 3 shows the accuracy of our R measurements. We measured the molecular ratio of a dry gas to a humid gas. The experiment was completed at room temperature (22–23 °C) and atmospheric pressure (1002–1006 hPa). The relative humidity of humid gases was measured. Figure 3a depicts R as a function of dew points calculated from the vapor pressure of water at these conditions. Errors of the measurements were ± 0.003 (0.3%, Fig. 3b).

### Effects of anesthetics and FIO2 on R

**Figure 4:**
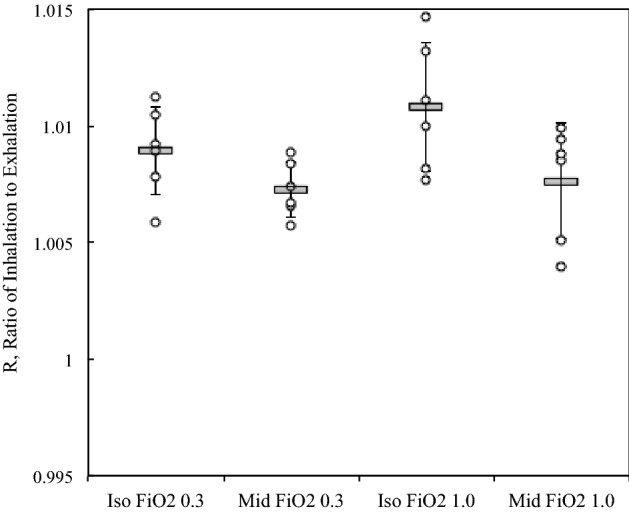
The results of R at each condition. The average R was 1.0099 ± 0.0023 with inhaled anesthetics (isoflurane), and it was 1.0074 ± 0.0018 with intravenous anesthetics (midazolam plus fentanyl). The average R was 1.0081 ± 0.0017 at an FIO2 of 0.3 and it was 1.0092 ± 0.0029 at an FIO2 of 1.0. There were no significant differences in the number of R from either anesthetics or FIO2.

Table 1 shows the results of multiple measurements of R for each condition. Measurements of R with the test lung were performed in every experiment to evaluate system performance. Since the test lung did not include gas exchange, R was expected to be 1.0. Our results support the high precision of our experiments with minimal day-to-day variability observed. The effects of anesthetics and FIO2 on R were tested in anesthetized rats. Figure 4 shows the results of R at each condition. The average R was 1.0099 ± 0.0023 with inhaled anesthetics (isoflurane), and it was 1.0074 ± 0.0018 with intravenous anesthetics (midazolam plus fentanyl). The average R was 1.0081 ± 0.0017 at an FIO2 of 0.3 and it was 1.0092 ± 0.0029 at an FIO2 of 1.0. There were no significant differences in R from either anesthetics or FIO2.

**Table 1:** (a) Midazolam/Fentanyl at FIO2 0.3, (b) Midazolam/Fentanyl at FIO2 1.0, (c) Isoflurane at FIO2 0.3, (d) Isoflurane at FIO2 1.0.

Measurement					Calculation
R-test lung	R-animal humid	Atmospheric pressure, hPa	Gas temp, °C	Gas RH, %	R-animal dry
**(a)**					
1.00118	0.98289	1002	22.7	91.7	1.00834
1.00118	0.98098	1002	22.8	91.6	1.00653
0.99903	0.97958	1007	23.3	91.5	1.00571
0.99903	0.98047	1003	23.4	90.7	1.00666
0.99643	0.98316	1020	23.1	91.8	1.00882
1.00195	0.98181	1012	23.3	89.9	1.00742
**(b)**					
1.00118	0.97981	1002	22.5	92.5	1.00510
1.00118	0.98382	1002	22.8	91.6	1.00944
0.99903	0.97780	1007	23.5	90.7	1.00397
0.99903	0.98420	1003	23.0	90.9	1.00990
0.99643	0.98330	1020	22.8	91.8	1.00849
1.00195	0.98302	1012	23.2	90.9	1.00879
**(c)**					
1.00120	0.98360	993	22.9	92.2	1.01045
1.00465	0.98146	1027	22.7	90.3	1.00586
0.99995	0.98297	1023	22.6	90.4	1.00784
1.00127	0.98366	1010	22.4	89.5	1.00923
1.00249	0.98329	1013	23.1	92.3	1.00896
1.00195	0.98526	1013	23.2	91.4	1.01121
**(d)**					
1.00120	0.98813	993	22.7	92.9	1.01465
1.00465	0.98288	1027	22.8	91.6	1.00768
0.99995	0.98532	1023	23.3	91.1	1.00999
1.00127	0.98338	1010	23.4	91.7	1.00817
1.00249	0.98546	1013	23.1	90.8	1.01107
1.00195	0.98723	1013	23.3	91.3	1.01320

### Effects of anesthetics and FIO2 on VCO2, VO2, and RQ

**Figure 5:**
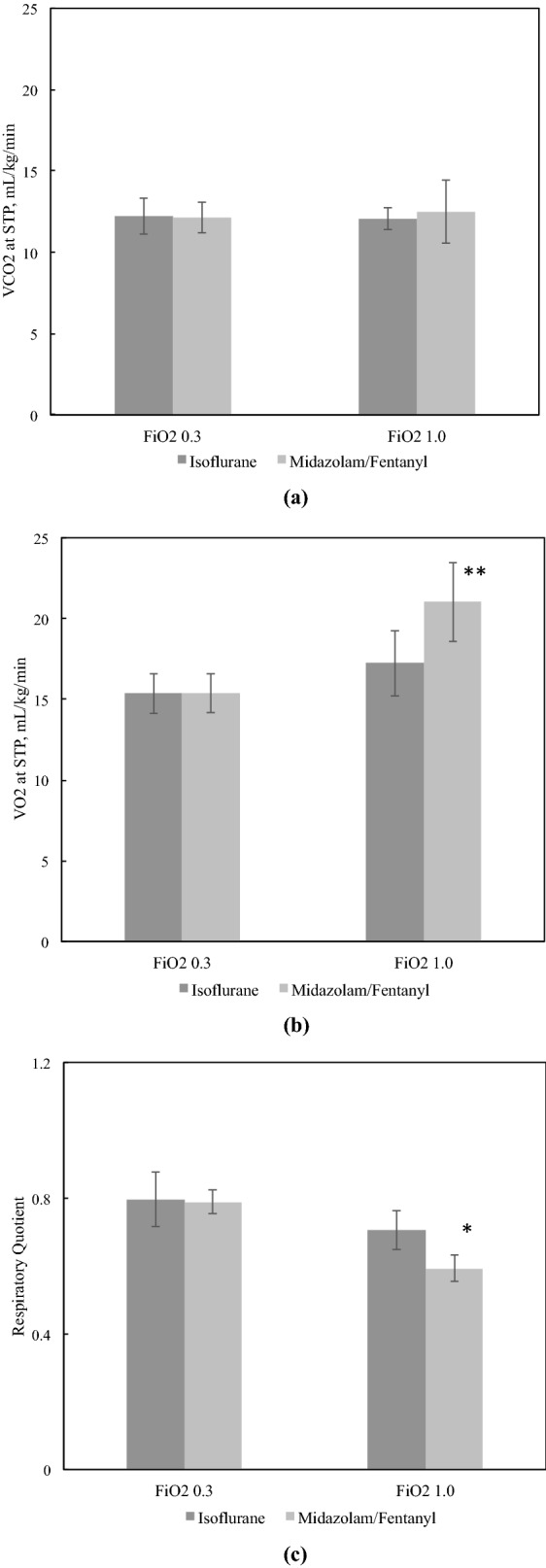
Effects of Anesthetics and FIO2 on VCO2, VO2, and RQ. (**a**) There were no significant differences in VCO2 between the FIO2 0.3 group and the FIO2 1.0 group. (**b**) VO2 increased at FIO2 1.0, which was more remarkable when intravenous anesthetics were used. (**c**) RQ of the intravenous anesthetic group was significantly lower at FIO2 1.0 than FIO2 0.3, while there was no significant change in the inhaled anesthetic group between FIO2 0.3 and FIO2 1.0. *, *p* < 0.05; **, *p* < 0.01.

The results are seen in Fig. 5. There were no significant differences in VCO2 (Fig. 5a) between the FIO2 0.3 group and the FIO2 1.0 group (isoflurane-FIO2 0.3: 12.2 ± 1.0; isoflurane-FIO2 1.0: 12.1 ± 0.6; midazolam/fentanyl-FIO2 0.3: 12.1 ± 0.8; midazolam/fentanyl‐FIO2 1.0: 12.5 ± 1.8 mL/kg per minute at standard temperature and pressure, respectively). However, VO2 (Fig. 5b) increased at FIO2 1.0, which was more remarkable when intravenous anesthetics were used (isoflurane-FIO2 0.3: 15.4 ± 1.1; isoflurane‐FIO2 1.0: 17.2 ± 1.8; midazolam/fentanyl-FIO2 0.3: 15.4 ± 1.1; midazolam/fentanyl‐FIO2 1.0: 21.0 ± 2.2 mL/kg per minute at standard temperature and pressure). As a result, the RQ (Fig. 5c) of the intravenous anesthetic group was significantly lower at FIO2 1.0 than FIO2 0.3, while there was no significant change in the inhaled anesthetic group between FIO2 0.3 and FIO2 1.0. (isoflurane-FIO2 0.3: 0.80 ± 0.07; isoflurane‐FIO2 1.0: 0.71 ± 0.05; midazolam/fentanyl-FIO2 0.3: 0.79 ± 0.03; midazolam/fentanyl‐FIO2 1.0: 0.59 ± 0.04).

### Effects of volume and pressure of ventilation on R

**Figure 6:**
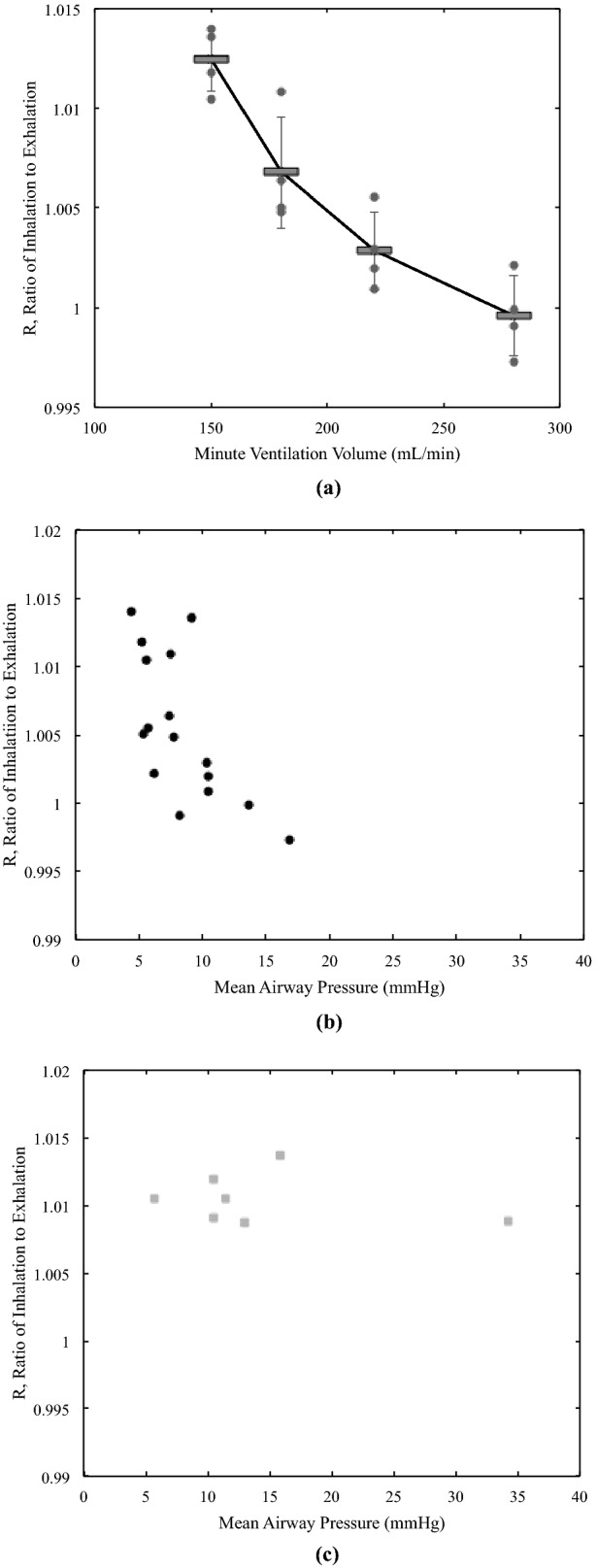
Effects of Volume and Pressure of Ventilation on R. (**a**) R is shown as a function of MVV (minute ventilation volume) ranged from 150 mL/min to 300 mL/min. R decreased to 1.0 as MVV increased. (**b**) The mean airway pressure changed from 4.4 to 16.8 mmHg as MVV increased from 150 to 280 mL/min and so there was a correlation of R with the airway pressure. (**c**) In a separate experimental setting at a fixed MVV of 180 mL/min, there were no significant changes in R at a range of the mean airway pressure from 5.7 to 34.2 mmHg.

Figure 6a depicts R as a function of MVV ranged from 150 to 300 mL/min. As shown in the figure, R decreased to 1.0 as MVV increased. The mean of R was 1.012 at an MVV of 150 mL/min, while it was 1.000 at an MVV of 280 mL/min. However, the mean airway pressure changed from 4.4 to 16.8 mmHg as MVV changed from 150 to 280 mL/min, and so there was a correlation of R with the airway pressure (Fig. 6b). Therefore, we conducted an experiment to test the effect of the airway pressure on R. At a fixed MVV of 180 mL/min, there were no significant changes in R at a range of mean airway pressure from 5.7 to 34.2 mmHg (Fig. 6c). This result supported that R depended on MVV but not on the airway pressure.

## Discussion

We developed a new method for measuring the molecular ratio of inhalation to exhalation that allows for an accurate metabolic measurement such as VCO2, VO2, and RQ in mechanically ventilated rats. We reported this novel measurement of the molecular ratio as R. Using our method, the effects of FIO2 on these metabolic parameters were tested. Our results suggest that VO2 increases at high FIO2 without changing the molecular ratio of inhalation to exhalation. This finding is novel and imperative to shed new lights on studies in oxygen metabolism of mammals. Because our method is non-invasive, the principle and the concept of our methods are applicable to indirect calorimetry for human beings.

The oxygen metabolism at high FIO2 has not been well described due to the lack of a reliable methodology. Lodato (1989)^19^ measured oxygen consumption during normobaric hyperoxia and decreased VO2 was reported at hyperoxia in dogs with the Fick method. However, Chapler (1984)^20^ found no effect of hyperoxia on VO2. A limited utility of the Fick method has been discussed due to its technical complexity^22^. Moreover, a major missing piece of this method is VCO2 or RQ, which is paramount in metabolic studies. Using calorimetry, we found that VO2 increased without an increase of VCO2 resulting in decreased RQ at high FIO2.

Our finding might be more remarkable in animals anesthetized with intravenous anesthetics, such as midazolam combined with fentanyl. We hypothesize that the endogenous oxidization of the anesthetics like midazolam and/or fentanyl, which reactions do not involve CO2 generation at oxidization, may contribute to the dissociation of VO2 from VCO2. Midazolam^23^ and fentanyl^24^ are metabolized in the liver with cytochrome P450 enzymes, which catalyze a variety of oxidation reactions without involvement of mitochondrial oxidative phosphorylation and Krebs cycle pathway that is the major source of CO2 production in mammals. Oxygen molecules are the substrates of these enzymes and so the reaction is O2 concentration dependent. We observed O2 concentration dependency of increased VO2 without a concomitant increase of VCO2 resulting in decreased RQ at an FIO2 of 1.0. This phenotype has not been well described in the previous studies but non-mitochondrial oxidase reactions could be a key to understand the mechanism of this unknown oxygen metabolism.

The other factor that can contribute to gas exchange is cutaneous respiration. It accounts for 2% of the lung respiration in humans^25^. The mechanism is diffusion and, in a condition that the skin has higher oxygen than atmosphere, the oxygen moves from the skin to the atmosphere rather than being absorbed into the skin and the subcutaneous tissues. At an FIO2 of 1.0, an oxygen concentration gradient of the skin surface is maximized and so the amount of oxygen that moves from the body surface to the atmosphere should be much higher than the numbers commonly acknowledged. Because there is no concentration gradient of carbon dioxide at FIO2 1.0, the diffusion mechanism contributes to increased VO2 but not VCO2. In this mode, oxygen is leaked rather than consumed. Both enzymatic reaction and diffusion mechanisms are O2 concentration dependent, which can be found in our results.

The adequacy of the Haldane transformation has been discussed over several decades^16,17^. There is an underlying fundamental question: is VI equal to VE? If the humidity of exhalation is included in the measurement, the answer for this question is clearly “no” since the vapor of water adds an amount of gas volume to VI and so VE becomes greater than VI. However, even if the humidity is appropriately removed from exhalation, the answer is likely “no” and VE seems smaller than VI.

The Haldane transformation is derived from the assumption that the volume of N2 inspired is equal to that expired:

10 $${\text{V}}_{{\text{I}}} /{\text{V}}_{{\text{E}}} = {\text{FEN}}2/{\text{FIN}}2$$

which is transformed to:

11 $${\text{V}}_{{\text{I}}} /{\text{V}}_{{\text{E}}} = \left( {1 - {\text{FEO}}2{-}{\text{FECO}}2} \right)/\left( {1{-}{\text{FIO}}2} \right).$$

Failure to account for the respiratory exchange ratio and assuming that VI equals VE have been cautioned^8^. The exchange ratio of O2 (FIO2 minus FEO2) is generally 5%, while FECO2 is lower and it is normally 4–4.5%. At room air (FIO2 0.21), therefore V_I_/V_E_ is expected to be 1.006 to 1.012. Interestingly, the number of R that we observed in this study ranged within these numbers. If V_I_/V_E_ is assumed to be 1.000, VO2 can be erroneously reported with lower numbers and yield error greater than 5%. All these discussions are based on the assumption that is expressed in Eqs. (10) and (11). Even though the conservation of N2 has been questioned^16,17^, the adequacy of the Haldane transformation has been supported for a long period.

One of the difficulties in this field is that we have not had a metric to know about the exact V̇ within an acceptable range of errors. Therefore, our new method of measuring the molecular ratio of inhalation to exhalation is of paramount importance. The benefit of our method is that it is an independent measurement from the gas concentrations. Based on our findings, the molecular ratio of inhalation to exhalation ranges from 1.007 to 1.010, which means VI is approximately 1% greater than VE. We do not have a clear explanation why VI is not equal to VE, however it is clear that the trend does not change when FIO2 increases, which is the novelty of this study, and it is inconsistent with the concept of the Haldane transformation.

One other important finding in this study is that R goes to 1.000 when MVV increases. The airway pressure correlated with it, however the actual independent factor that changed R was the ventilation volume. This suggests that the change of the molecular ratio may be still related to the gas exchange. RQ is known to increase at hyperventilation^4^. When RQ goes to 1.0 while hyperventilating the subject^26^, there may be a chance of the molecular ratio becoming 1.000. However, this interpretation follows the concept of Eq. (11) and so that of the Haldane transformation. FIO2 does not affect R but gas exchange may affect R. This raises a possibility that we are missing some components in Eq. (11) that adjusts the denominator when high FIO2 is used.

The study limitation is that we did not have a gold standard method that enabled us to compare with the methods. However, there is currently no commercial device available that measures VO2 in rodents that are on mechanical ventilation. We assembled commercially available sensors and validated errors in our experimental setting. Our oxygen sensor had the error range of ± 0.2% between the range of 21–100%. As it is described in the manuscript, that of R was ± 0.3%. These were the random errors that occurred independently. Since we did not change the ventilation volume, VE is considered a constant value. Therefore, the error range of VO2 that is calculated from the Eq. (7) is calculated as ± 7.5% at FIO2 1.0 and  ± 2.5% at FIO2 0.3. Further validation study is required as the error range may vary based on each experimental condition, however we consider that our error range is acceptable to evaluate the effect of high FIO2 on VO2 in our rat experimental model and adequately draw the conclusions.

## Conclusion

Using calorimetry and a new method for measuring the molecular ratio of inhalation to exhalation, VO2 increased without a concomitant increase of VCO2 at an FIO2 of 1.0, and as a result RQ decreased in mechanically ventilated rats.

## Supplementary Information

Supplementary Figures.
