# Supplementary material for: A method for measuring the molecular ratio of inhalation to exhalation and effect of inspired oxygen levels on oxygen consumption
Source: Sci Rep. 2021 Jun 17;11:12815. doi: 10.1038/s41598-021-91246-8 (PMC8211831; doi:10.1038/s41598-021-91246-8)
Supplement: Supplementary file 1 — Supplementary Figures. [file 41598_2021_91246_MOESM1_ESM.pptx]

## Slide 1
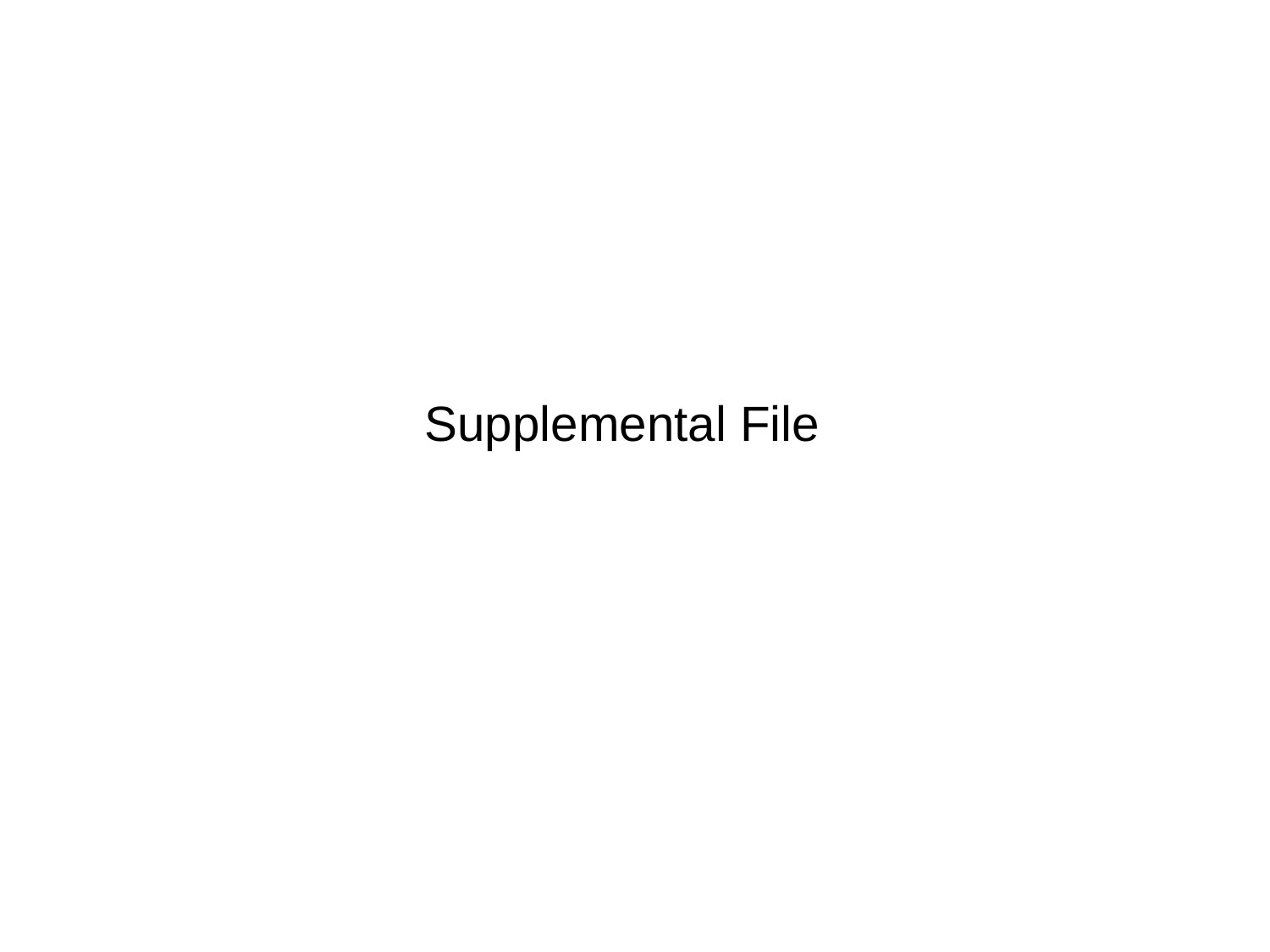

Supplemental File

## Slide 2
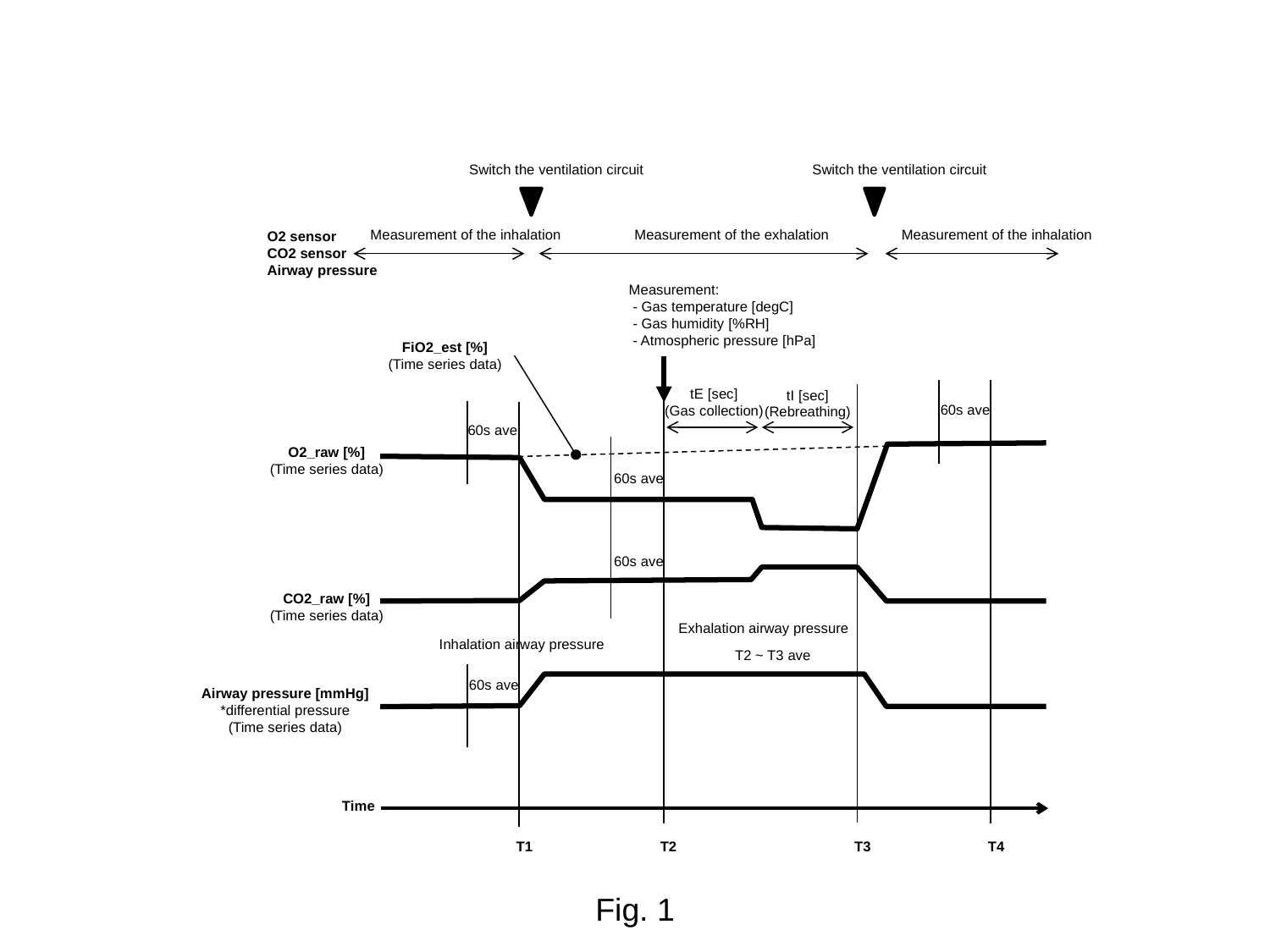

Switch the ventilation circuit
Switch the ventilation circuit
Measurement of the inhalation
Measurement of the exhalation
Measurement of the inhalation
O2 sensor
CO2 sensor
Airway pressure
Measurement:
 - Gas temperature [degC]
 - Gas humidity [%RH]
 - Atmospheric pressure [hPa]
FiO2_est [%]
(Time series data)
tE [sec]
(Gas collection)
tI [sec]
(Rebreathing)
60s ave
60s ave
O2_raw [%]
(Time series data)
60s ave
60s ave
CO2_raw [%]
(Time series data)
Exhalation airway pressure
Inhalation airway pressure
T2 ~ T3 ave
60s ave
Airway pressure [mmHg]
*differential pressure
(Time series data)
Time
T1
T2
T3
T4
Fig. 1

## Slide 3
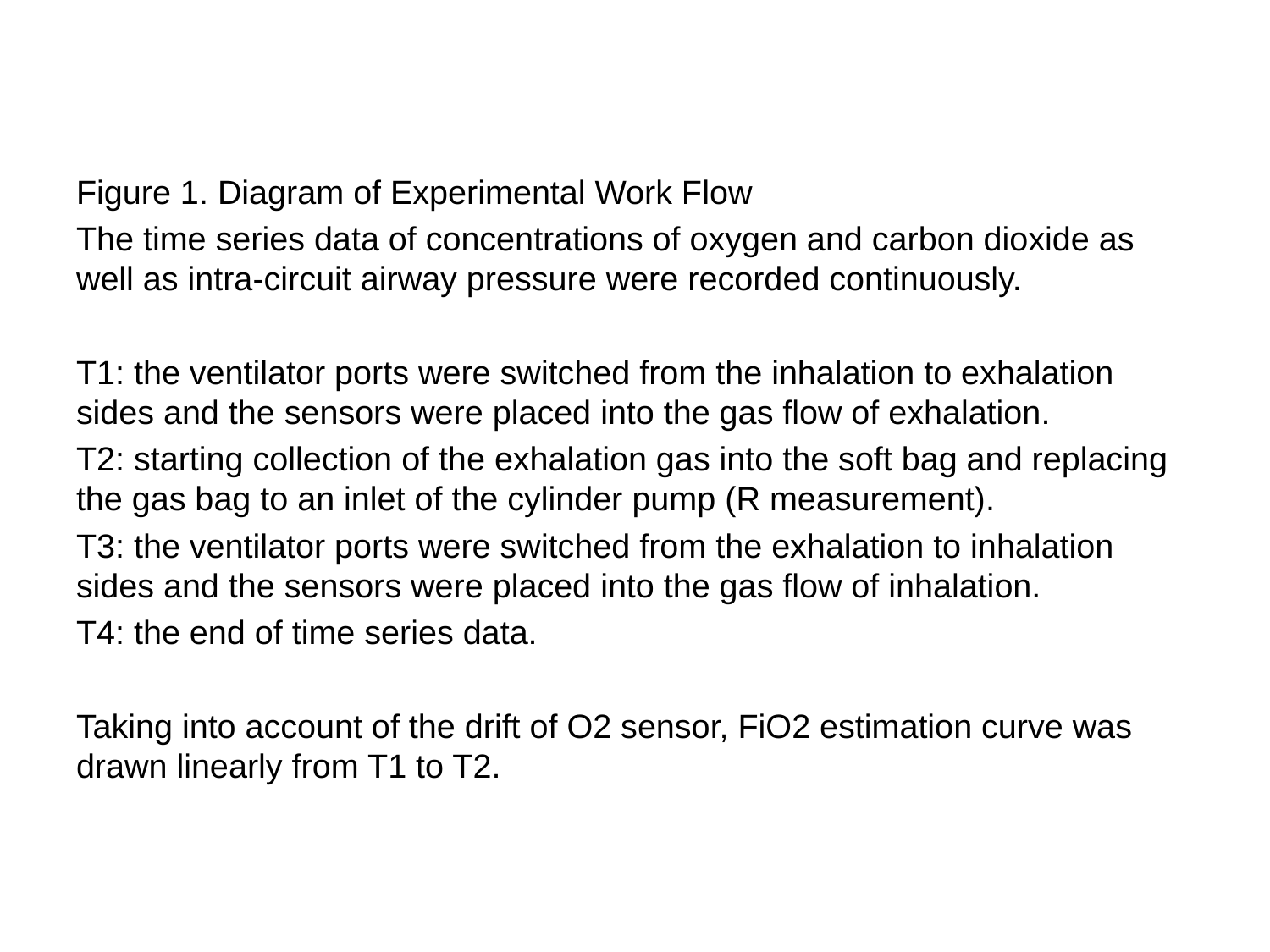

Figure 1. Diagram of Experimental Work Flow
The time series data of concentrations of oxygen and carbon dioxide as well as intra-circuit airway pressure were recorded continuously.
T1: the ventilator ports were switched from the inhalation to exhalation sides and the sensors were placed into the gas flow of exhalation.
T2: starting collection of the exhalation gas into the soft bag and replacing the gas bag to an inlet of the cylinder pump (R measurement).
T3: the ventilator ports were switched from the exhalation to inhalation sides and the sensors were placed into the gas flow of inhalation.
T4: the end of time series data.
Taking into account of the drift of O2 sensor, FiO2 estimation curve was drawn linearly from T1 to T2.

## Slide 4
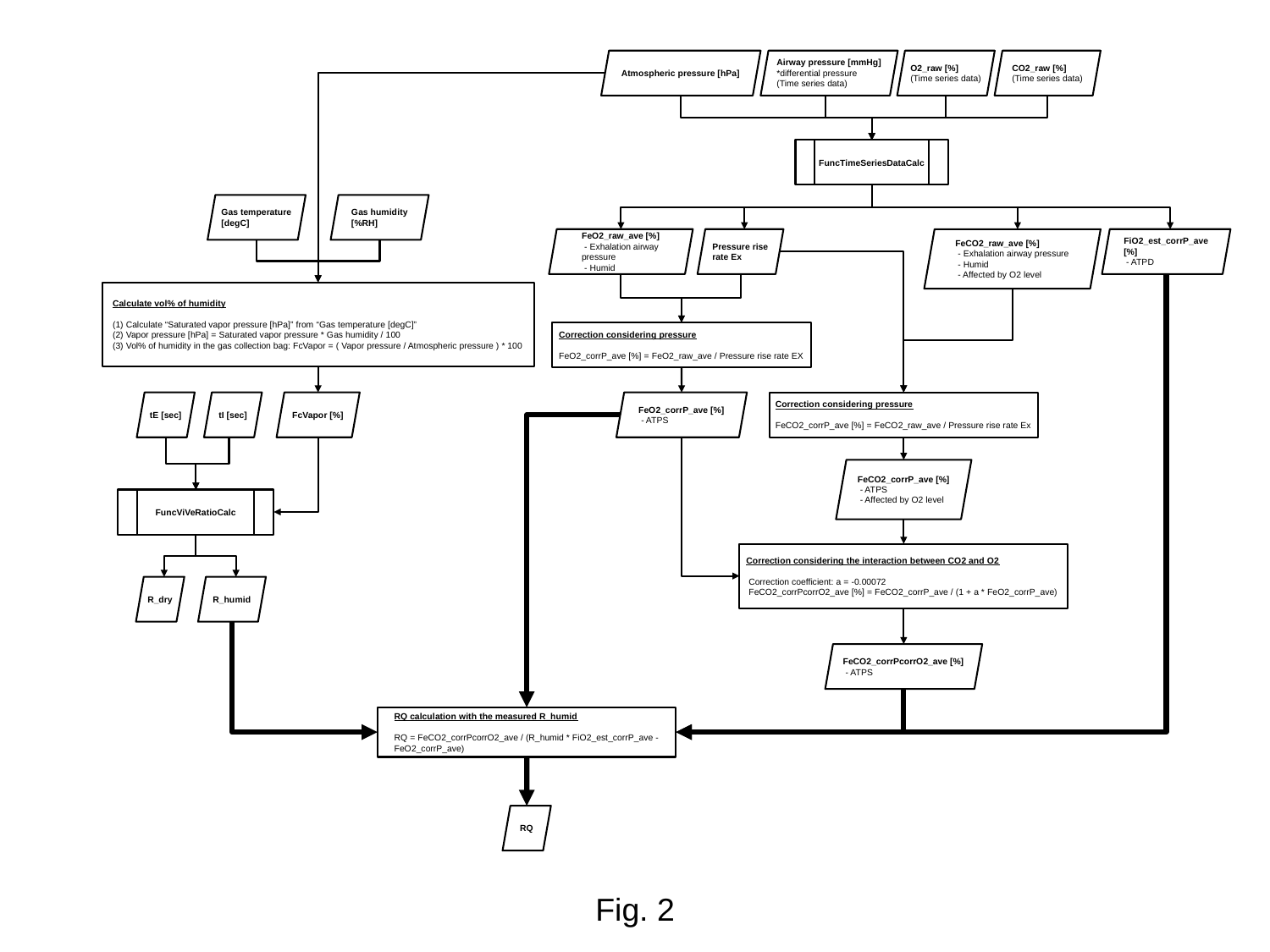

Atmospheric pressure [hPa]
Airway pressure [mmHg]
*differential pressure
(Time series data)
O2_raw [%]
(Time series data)
CO2_raw [%]
(Time series data)
FuncTimeSeriesDataCalc
Gas temperature [degC]
Gas humidity [%RH]
FeCO2_raw_ave [%]
 - Exhalation airway pressure
 - Humid
 - Affected by O2 level
FeO2_raw_ave [%]
 - Exhalation airway pressure
 - Humid
Pressure rise rate Ex
FiO2_est_corrP_ave [%]
 - ATPD
Calculate vol% of humidity
(1) Calculate “Saturated vapor pressure [hPa]” from “Gas temperature [degC]”
(2) Vapor pressure [hPa] = Saturated vapor pressure * Gas humidity / 100
(3) Vol% of humidity in the gas collection bag: FcVapor = ( Vapor pressure / Atmospheric pressure ) * 100
Correction considering pressure
FeO2_corrP_ave [%] = FeO2_raw_ave / Pressure rise rate EX
Correction considering pressure
FeCO2_corrP_ave [%] = FeCO2_raw_ave / Pressure rise rate Ex
tE [sec]
tI [sec]
FcVapor [%]
FeO2_corrP_ave [%]
 - ATPS
FeCO2_corrP_ave [%]
 - ATPS
 - Affected by O2 level
FuncViVeRatioCalc
Correction considering the interaction between CO2 and O2
 Correction coefficient: a = -0.00072
 FeCO2_corrPcorrO2_ave [%] = FeCO2_corrP_ave / (1 + a * FeO2_corrP_ave)
R_dry
R_humid
FeCO2_corrPcorrO2_ave [%]
 - ATPS
RQ calculation with the measured R_humid
RQ = FeCO2_corrPcorrO2_ave / (R_humid * FiO2_est_corrP_ave - FeO2_corrP_ave)
RQ
Fig. 2

## Slide 5
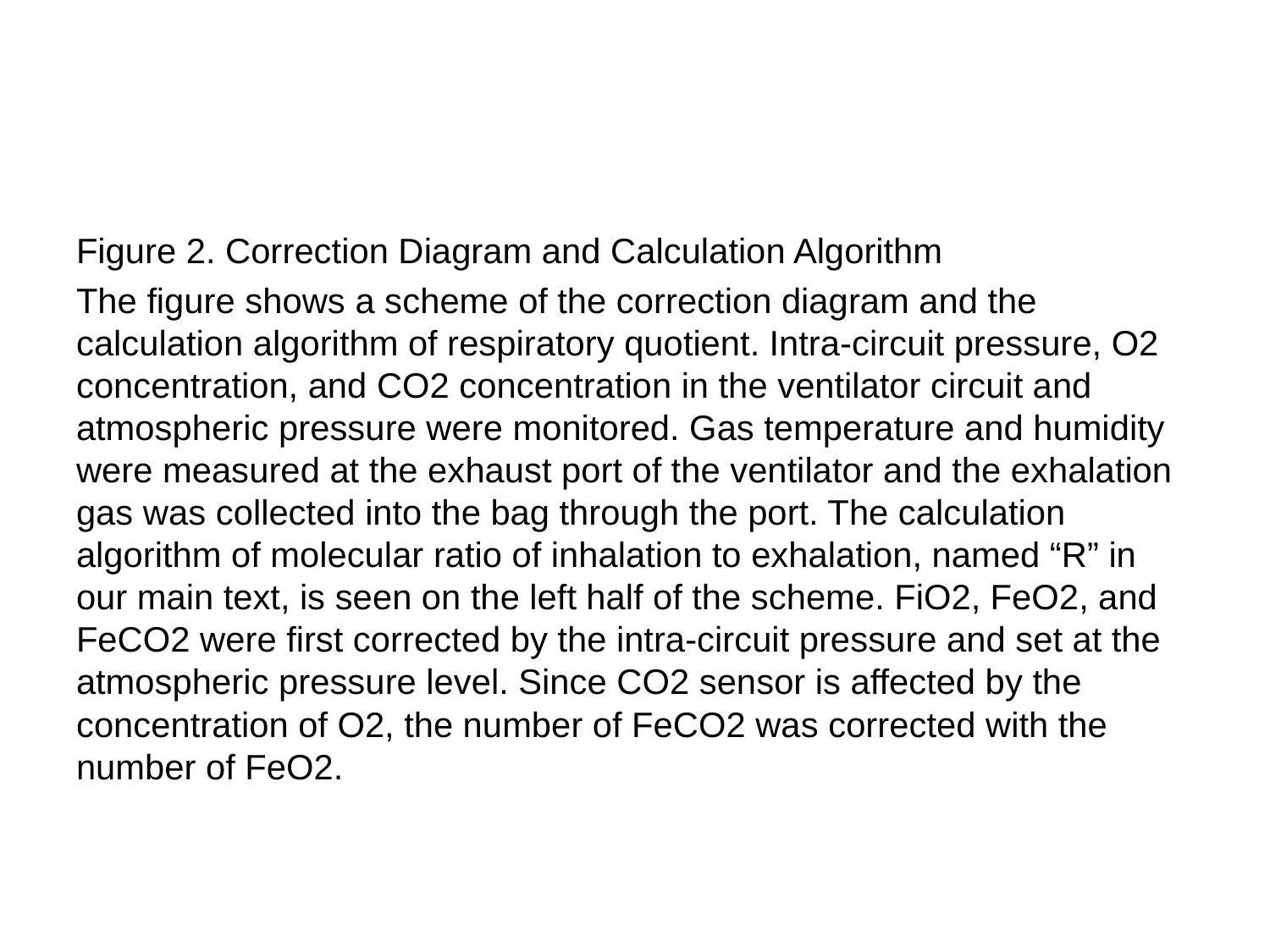

Figure 2. Correction Diagram and Calculation Algorithm
The figure shows a scheme of the correction diagram and the calculation algorithm of respiratory quotient. Intra-circuit pressure, O2 concentration, and CO2 concentration in the ventilator circuit and atmospheric pressure were monitored. Gas temperature and humidity were measured at the exhaust port of the ventilator and the exhalation gas was collected into the bag through the port. The calculation algorithm of molecular ratio of inhalation to exhalation, named “R” in our main text, is seen on the left half of the scheme. FiO2, FeO2, and FeCO2 were first corrected by the intra-circuit pressure and set at the atmospheric pressure level. Since CO2 sensor is affected by the concentration of O2, the number of FeCO2 was corrected with the number of FeO2.

## Slide 6
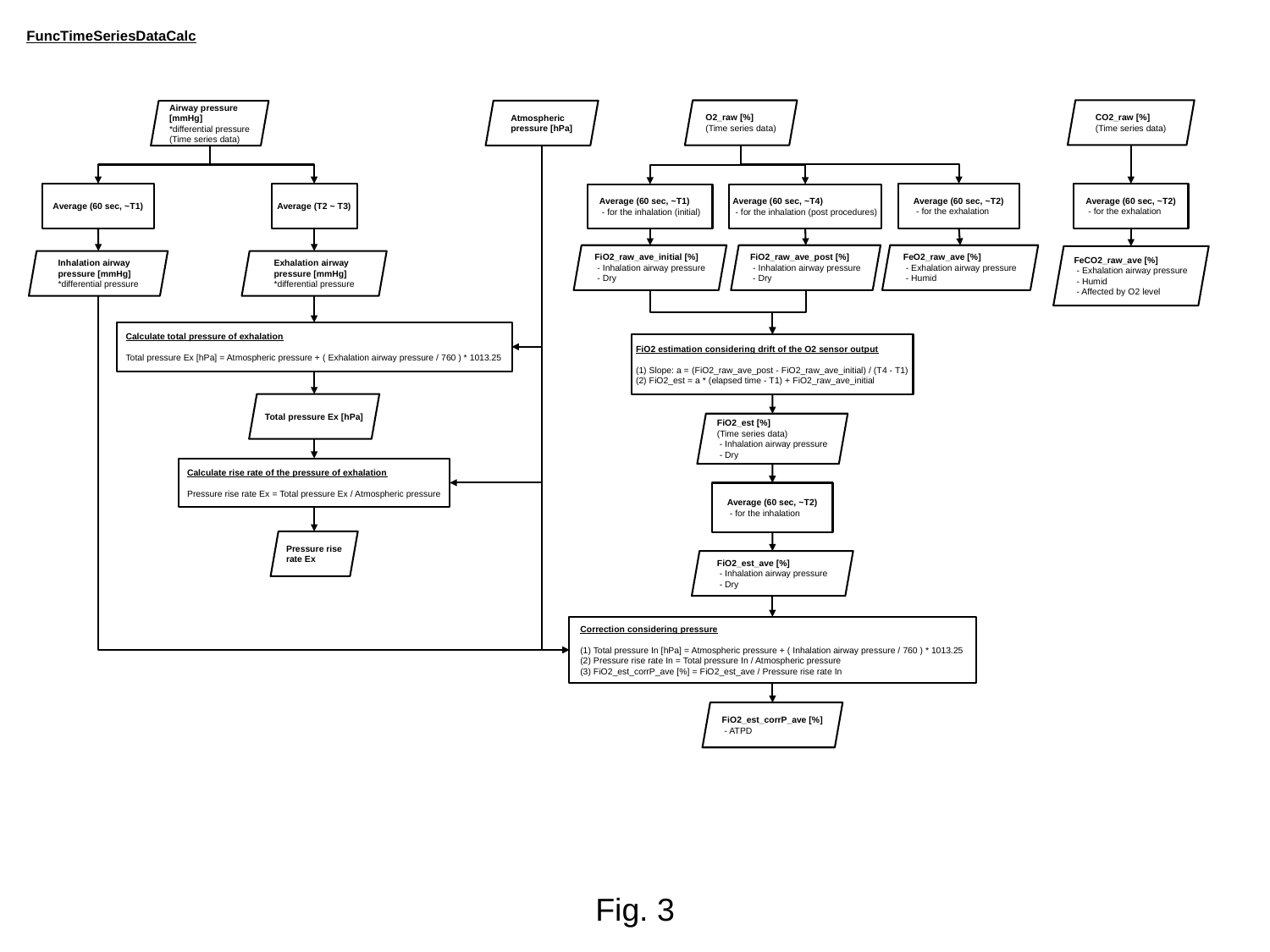

FuncTimeSeriesDataCalc
CO2_raw [%]
(Time series data)
O2_raw [%]
(Time series data)
Atmospheric pressure [hPa]
Airway pressure [mmHg]
*differential pressure
(Time series data)
Average (60 sec, ~T2)
 - for the exhalation
Average (60 sec, ~T2)
 - for the exhalation
Average (60 sec, ~T1)
Average (T2 ~ T3)
Average (60 sec, ~T1)
 - for the inhalation (initial)
Average (60 sec, ~T4)
 - for the inhalation (post procedures)
FiO2_raw_ave_initial [%]
 - Inhalation airway pressure
 - Dry
FiO2_raw_ave_post [%]
 - Inhalation airway pressure
 - Dry
FeO2_raw_ave [%]
 - Exhalation airway pressure
 - Humid
FeCO2_raw_ave [%]
 - Exhalation airway pressure
 - Humid
 - Affected by O2 level
Inhalation airway pressure [mmHg]
*differential pressure
Exhalation airway pressure [mmHg]
*differential pressure
Calculate total pressure of exhalation
Total pressure Ex [hPa] = Atmospheric pressure + ( Exhalation airway pressure / 760 ) * 1013.25
FiO2 estimation considering drift of the O2 sensor output
(1) Slope: a = (FiO2_raw_ave_post - FiO2_raw_ave_initial) / (T4 - T1)
(2) FiO2_est = a * (elapsed time - T1) + FiO2_raw_ave_initial
Total pressure Ex [hPa]
FiO2_est [%]
(Time series data)
 - Inhalation airway pressure
 - Dry
Calculate rise rate of the pressure of exhalation
Pressure rise rate Ex = Total pressure Ex / Atmospheric pressure
Average (60 sec, ~T2)
 - for the inhalation
Pressure rise rate Ex
FiO2_est_ave [%]
 - Inhalation airway pressure
 - Dry
Correction considering pressure
(1) Total pressure In [hPa] = Atmospheric pressure + ( Inhalation airway pressure / 760 ) * 1013.25
(2) Pressure rise rate In = Total pressure In / Atmospheric pressure
(3) FiO2_est_corrP_ave [%] = FiO2_est_ave / Pressure rise rate In
FiO2_est_corrP_ave [%]
 - ATPD
Fig. 3

## Slide 7
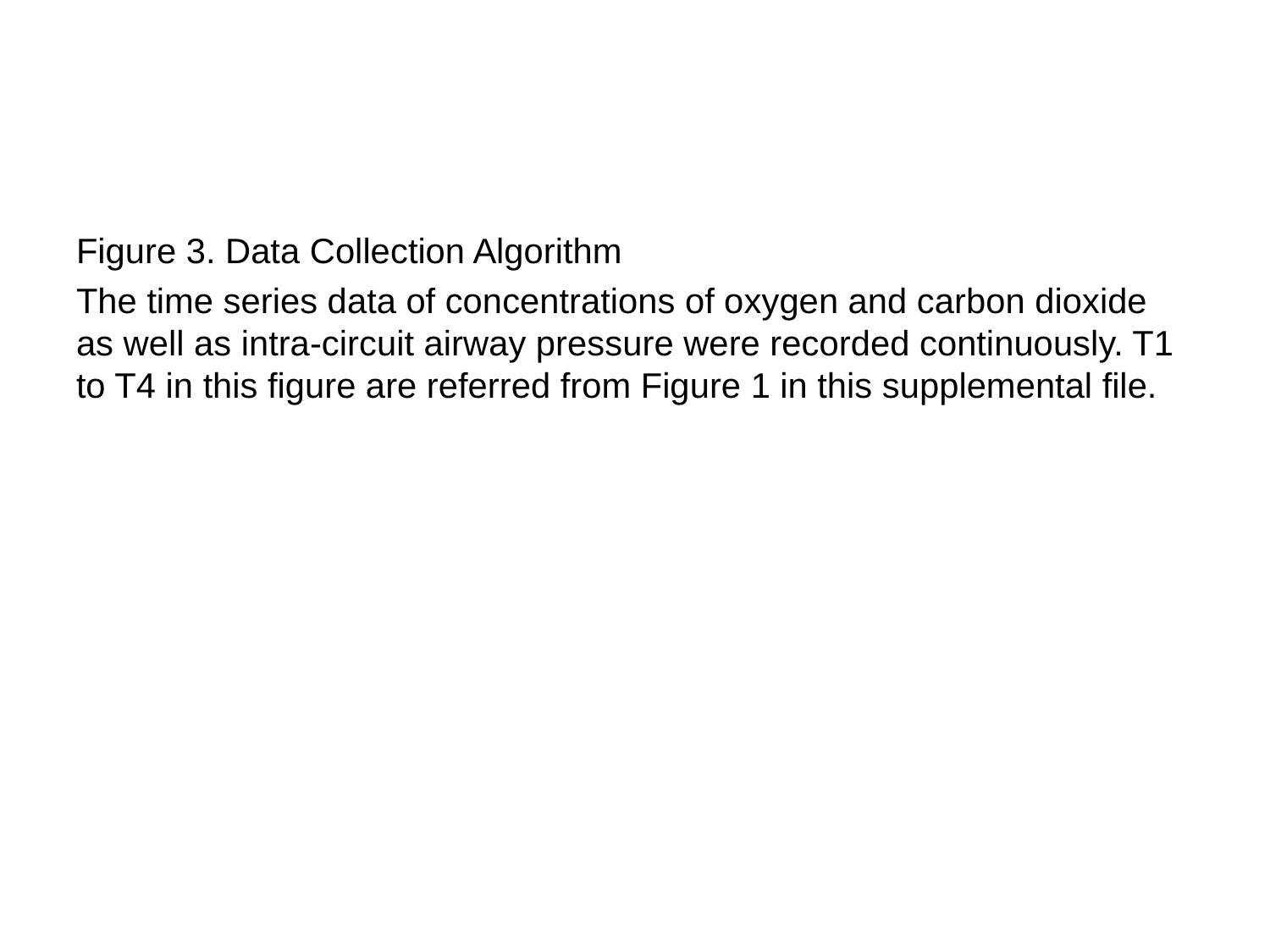

Figure 3. Data Collection Algorithm
The time series data of concentrations of oxygen and carbon dioxide as well as intra-circuit airway pressure were recorded continuously. T1 to T4 in this figure are referred from Figure 1 in this supplemental file.

## Slide 8
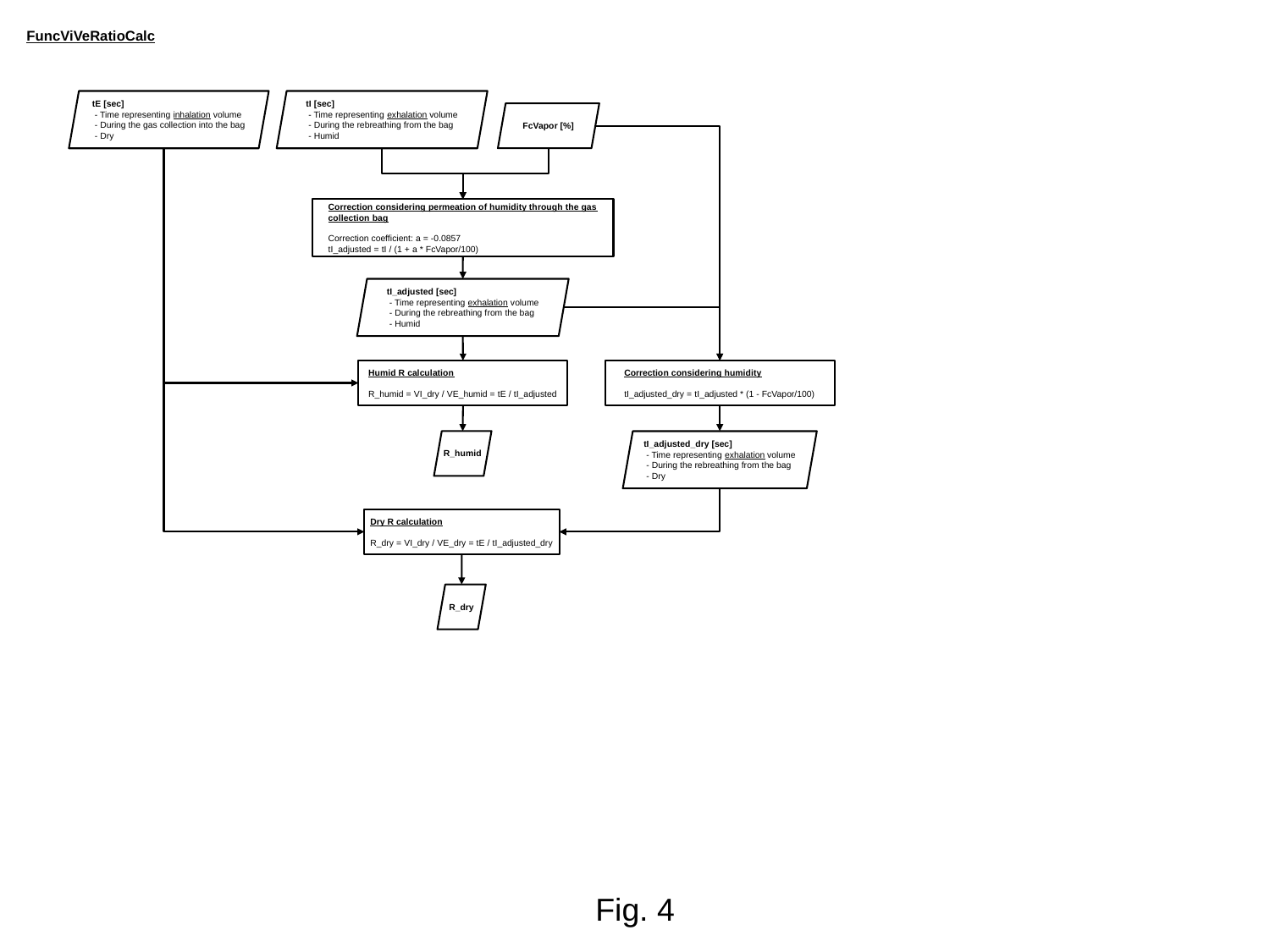

FuncViVeRatioCalc
tE [sec]
 - Time representing inhalation volume
 - During the gas collection into the bag
 - Dry
tI [sec]
 - Time representing exhalation volume
 - During the rebreathing from the bag
 - Humid
FcVapor [%]
Correction considering permeation of humidity through the gas collection bag
Correction coefficient: a = -0.0857
tI_adjusted = tI / (1 + a * FcVapor/100)
tI_adjusted [sec]
 - Time representing exhalation volume
 - During the rebreathing from the bag
 - Humid
Correction considering humidity
tI_adjusted_dry = tI_adjusted * (1 - FcVapor/100)
Humid R calculation
R_humid = VI_dry / VE_humid = tE / tI_adjusted
R_humid
tI_adjusted_dry [sec]
 - Time representing exhalation volume
 - During the rebreathing from the bag
 - Dry
Dry R calculation
R_dry = VI_dry / VE_dry = tE / tI_adjusted_dry
R_dry
Fig. 4

## Slide 9
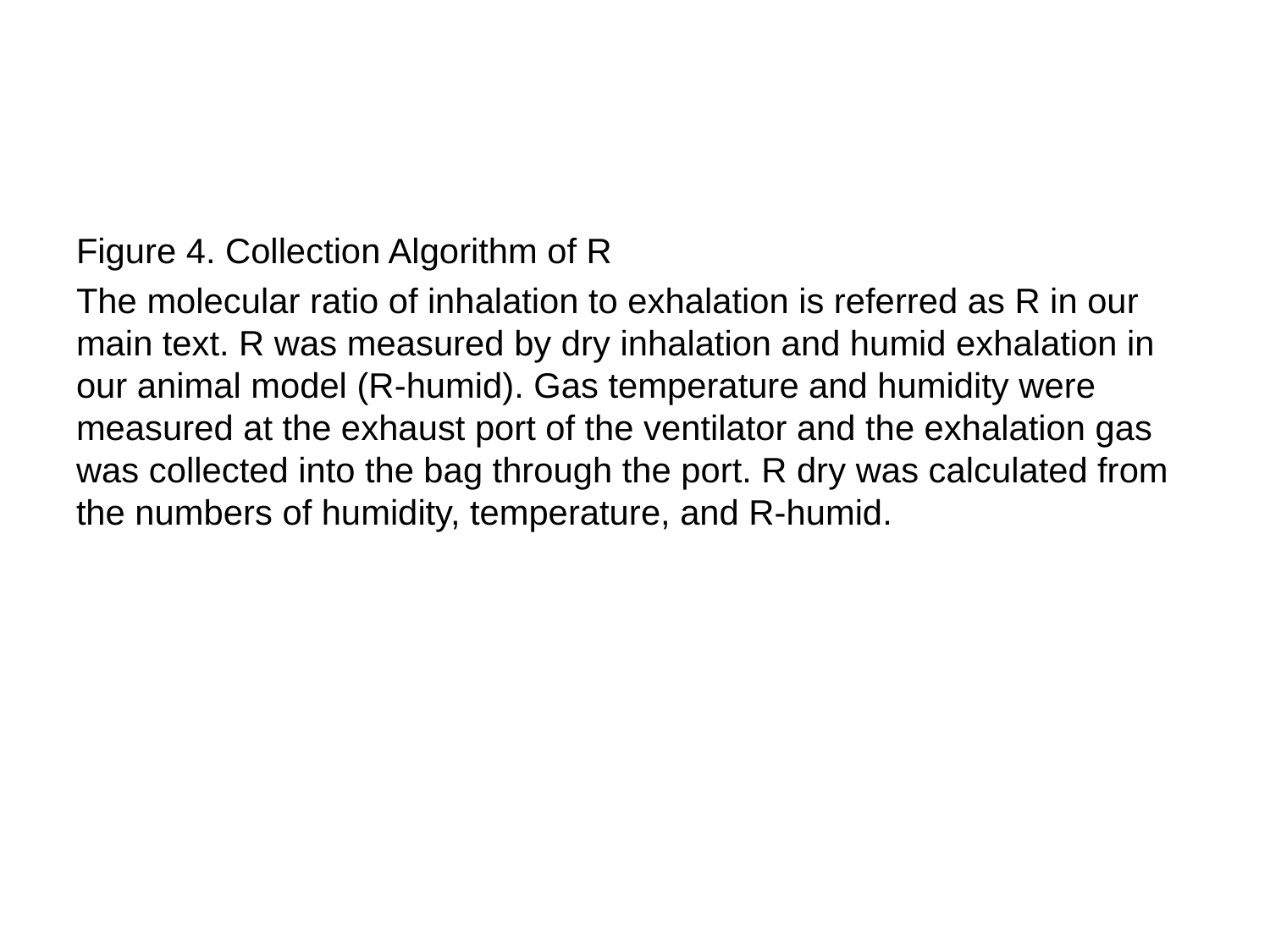

Figure 4. Collection Algorithm of R
The molecular ratio of inhalation to exhalation is referred as R in our main text. R was measured by dry inhalation and humid exhalation in our animal model (R-humid). Gas temperature and humidity were measured at the exhaust port of the ventilator and the exhalation gas was collected into the bag through the port. R dry was calculated from the numbers of humidity, temperature, and R-humid.
